# Molecular Characterization and Phylogenetic Analysis of Hepatitis E Virus (HEV) Strains from Pigs Farmed in Eight European Countries between 2020 and 2022

**DOI:** 10.1155/2023/2806835

**Published:** 2023-12-07

**Authors:** Luca De Sabato, Giovanni Ianiro, Giovanni L. Alborali, Annelies Kroneman, Sylvia S. Grierson, Gergana Lyubomirova Krumova-Valcheva, Renate W. Hakze-van der Honing, Reimar Johne, Ivana Kolackova, Iwona Kozyra, Eva Gyurova, Enrico Pavoni, Katharina Reisp, Elena Lucia Sassu, Katja Schilling-Loeffler, Richard Piers Smith, Petra Vasickova, Jacek Żmudzki, Artur Rzeżutka, Ilaria Di Bartolo

**Affiliations:** ^1^Department Food Safety, Nutrition and Veterinary public Health, Istituto Superiore di Sanità, Rome, Italy; ^2^Istituto Zooprofilattico Sperimentale della Lombardia e dell'Emilia-Romagna “Bruno Ubertini”, Brescia, Italy; ^3^Centre for Infectious Disease Control, National Institute for Public Health and the Environment (RIVM), Bilthoven, Netherlands; ^4^Animal and Plant Health Agency, Weybridge, Addlestone, UK; ^5^National Diagnostic and Research Veterinary Medicine Institute, National Food Safety Centre, Sofia 1606, Bulgaria; ^6^Wageningen University and Research, Houtribweg 39, 8221 RA, Lelystad, Netherlands; ^7^German Federal Institute for Risk Assessment, Max-Dohrn-Strasse 8-10, 10589, Berlin, Germany; ^8^Department of Public Health, Masaryk University, Brno, Czech Republic; ^9^Department of Food and Environmental Virology, National Veterinary Research Institute, Al. Partyzantów 57, 24-100, Puławy, Poland; ^10^Division for Animal Health, Austrian Agency for Health and Food Safety (AGES), Mödling, Austria; ^11^Laboratory of Neurobiology and Pathological Physiology, Institute of Animal Physiology and Genetics, The Czech Academy of Sciences, Brno, Czech Republic; ^12^Department of Swine Diseases, National Veterinary Research Institute, Al. Partyzantów 57, Puławy 24-100, Poland

## Abstract

In high-income countries, the hepatitis E virus (HEV) is considered an emerging threat causing autochthonous acute hepatitis in humans, with an increased number of reported cases over the last 10 years and related increased burden of chronic hepatitis in immunocompromised and transplant patients. Pigs are the main reservoir of the HEV-3 genotype, which is the most common in Europe, and can be transmitted to humans through the consumption of raw and undercooked pork products. Extensive sequencing revealed the existence of several HEV-3 subtypes in both humans and pigs, confirming a broad heterogeneity of the virus, with some subtypes, such as 3e, 3f, and 3c, which are predominant in Europe. In this study, 291 HEV sequences were obtained from pig feces sampled in more than 74 farms located in Austria, Bulgaria, Czech Republic, Germany, Italy, Poland, the United Kingdom, as well as an unknown number of farms in Netherlands. Of the 99 nonidentical sequences (99/291), 90 were assigned to seven established HEV-3 subtypes: 3a, 3c, 3e, 3f, 3g (here named 3g-like), 3i, and 3l (named 3l-like), already described in Europe, while nine sequences of HEV-3 could not be assigned to any existing subtype (here named 3 ^*∗*^). The 3e subtype was the most common, detected in six out of eight countries, followed by 3f and 3c, which were also present in several countries; 3g-like, 3i, and 3l-like subtypes showed only a limited circulation. The distribution of frequently (3e, 3f, and 3c) and rarely (3g-like, 3i, and 3l-like) detected HEV-3 subtypes in pigs was correlated with their detection rates in human patients in Europe. The results from this study confirm the wide circulation of several HEV-3 strains in European pigs and confirm that sequencing is needed to monitor the different strains and to identify possible zoonotic transmission paths.

## 1. Introduction

The hepatitis E virus (HEV), classified as Paslahepevirus balayani within the *Hepeviridae* family, is considered an emerging public health threat worldwide. HEV infections may have different clinical course ranging from asymptomatic cases to acute hepatitis, mainly self-limiting, but occasionally fulminant and chronic in immunocompromised patients [1, 2].

The World Health Organization (WHO) estimates approximately 20 million HEV infections every year, with 3 million symptomatic manifestations and almost 60,000 HEV-related deaths (https://www.who.int/news-room/fact-sheets/detail/hepatitis-e, accessed on 25th October, 2023).

Within the *Hepeviridae* family, the *Paslahepevirus* genus includes four main HEV genotypes with strains infecting mammals, in particular humans and several animal species, with two main transmission patterns linked to specific epidemiological distributions [3, 4]. Genotypes HEV-1 and HEV-2 infect only humans and circulate mainly in low-income countries, causing large outbreaks due to the consumption of contaminated drinking water. Genotypes HEV-3 and HEV-4 are zoonotic and widely distributed in high-income countries, where they typically cause both sporadic cases and confined outbreaks [5]. The first animal strain of HEV was described in domestic pigs in 1997 in the United States and was called swine HEV (sHEV) [6]. Following sequence analyses revealed that the swine HEV-3 strain was similar to human HEV-3 strains. Since then, HEV-3 strains had been reported in pigs worldwide [7–14]. The other zoonotic genotype HEV-4 was first circulating in Asia and recently rarely detected also in pigs [15, 16] and humans in Europe [17, 18]. Pigs are now recognized as the main reservoir of the zoonotic HEV [19].

The main transmission route of HEV-3 and HEV-4 is foodborne. The occurrence of HEV-3 and HEV-4 infections in humans has been frequently linked to the consumption of HEV contaminated raw or uncooked pork and wild boar products mainly containing liver, which is the target organ of virus replication. In Europe, HEV-3 is the most common circulating genotype in both humans and pigs [5].

Studies of foodborne outbreaks proved the source of infection in meat products, mainly pork liver sausages, deer sashimi, and wild boar meat, by identifying identical sequences in both patients and leftover food [5, 20]. Epidemiological studies also confirmed the role of meat products as the source of infections by linking the consumption of pork with an increased risk of either HEV-3 or HEV-4 infections [5].

From 2005 to 2015, an increasing number of human cases were reported in the EU, which could have been due to a higher clinicians' awareness [20, 21]. The HEV-3 prevalence in the EU domestic pigs ranges between 2.5% [22] and 49.5% [23] on farms and between 5.0% and 11.0% at the slaughterhouse [19, 24]. To date, it is difficult to compare the results in virus prevalence, due to different type and number of samples analyzed and to different methods applied for HEV detection [19].

Based on the analyses of full genome sequences, HEV-3 strains have been classified into 19 subtypes, 13 assigned as HEV-3a to 3m and six still provisionally classified [25]. The frequency of these different subtypes is variable, with the 3c, 3e, and 3f as the most common in Europe, both in humans and animals [26–28]. Other subtypes are either more common in wild boars than in pigs or present a lower detection rate in pigs [29].

Over the last decade, in Europe, a change in the subtype circulation in humans has been observed with an increase of 3c strains compared to a previous large circulation of 3e and 3f subtypes. This change was observed in France [28, 30], England [31, 32], Belgium [33], Germany [34], and Netherlands [35]. In other countries, such as Italy and Spain [36], the 3e and 3f subtypes are still predominant [37, 38]. Recent studies suggested differences in the pathogenicity of the subtypes [34, 39], but results are still debate since some others do not remark the same correlation [40].

The aim of the present study was to characterize the HEV strains circulating in domestic pigs at farms in eight European countries and to assess the correlation between subtype detection in swine, wild boar, and human across Europe. The molecular data obtained will improve the knowledge on both the geographical spread and the routes of transmission of viral strains among European countries.

## 2. Materials and Methods

### 2.1. Sample Collection for Sequencing

Two hundred ninety-one HEV-positive fecal samples were collected from domestic pigs from February 2020 to January 2022 (*Supplementary 1*). Each farm was sampled by collecting 20 samples on the same day, and each sample was a pooled sample consisting of 10 individual feces. Samples were analyzed for the detection of HEV in the framework of the BIOPIGEE project (European Union's Horizon 2020 research and innovation programme under grant agreement no. 773830), from eight European countries: Austria, Bulgaria, Czech Republic, Germany, Italy, Netherlands, Poland, and the United Kingdom. Overall, 74 farms in seven countries were found to be HEV positive, for Netherlands information on farms was not available (Table 1). Samples were chosen among HEV-positive specimens with low Ct values (<30) obtained by the previously performed real-time RT-PCR used for the virus detection [41]. From each farm, 1–10 HEV sequences were obtained for an overall number of 291 sequences from more than 74 farms (Table 1). The identification of the genotype and subtypes of HEV strains was based on the phylogenetic analysis of the HEV genome fragment (493 bp) within the ORF2 region [12]. The 291 obtained amplicons were sequenced in both directions [12] using Sanger sequencing method and submitted to the HEV-Net [42] and the NCBI database, under the following accession numbers: OQ595015 to OQ595079 and from OQ686829 to OQ686862.

### 2.2. Identification of HEV Subtypes

The first step was the identification of identical sequences from the same farm. The sequences alignment was performed using MAFFT [43] and manually edited with AliView [44]. The maximum likelihood phylogenetic tree was run with 1,000 bootstrap replicates in IQ-TREE version 2.2.0 software [45]. The phylogenetic trees were modified, adding a heatmap with host and country information for each tree using R software version 4.1.2 [46] and the ggtree package [47]. The proportion (p) of nucleotide sites at which two sequences being compared are different (*p*-distance values) was calculated using MEGAX software [48]. Identical sequences from the same farm were excluded from the following analyses, performed on a final number of nonidentical sequences of 99.

The second step was the attribution of subtypes to each of the 99 nonidentical sequences. To this purpose, a phylogenetic tree was built, following the method described above, using nonidentical sequences (*n* = 99) and the HEV-3 subtypes reference sequences (*n* = 18), including 12 assigned to 3a–3m subtypes, and six HEV-3 reference sequences still unassigned to any subtype [25]. The genotype HEV-4 sequence was used as outgroup (accession number: LC022745).

The subtype attribution of sequences was also performed using the “HEV-Net database typing tool version 1.0” (https://www.rivm.nl/mpf/typingtool/hev/, accessed on 26th October, 2023).

### 2.3. Phylogenetic Analyses of Pig HEV Sequences

Afterward, seven datasets, one for each subtype identified in this study, were built to perform phylogenetic analysis and compare the newly obtained pig sequences with HEV sequences of human and animal origin available online in the NCBI database.

The steps described below were automized with scripts written in Python 3 [49]. HEV sequences were selected with three criteria: (1) sequence length >250 bp, with information available on (2) country and (3) host. The download of the HEV sequences from NCBI database (accessed on October 2022) following the criteria reported above was performed using the Biopython library [50] and the commands Entrez.esearch and Entrez.efetch using the nuccore as database and the term ““hepatitis e” AND “host” (ALL) AND “country” (ALL) AND (“250” (SLEN) : “10,000,000” (SLEN)).” The whole database resulted in 14.749 sequences and used to compare with each subtype dataset built with pig HEV sequences generated in this study. The BLASTN algorithm (Megablast) was used for the comparison. The sequences retrieved were further subsampled by a two-stage process to build the final dataset to draw the phylogenetic trees. Sequences displaying >91.0% nucleotide identity with those of this study were selected in the first stage of the selection. In the second stage, to build the dataset, 10.0% of sequences with an identity between 91.0% and 98.0% were selected by random subsampling according to country and host information. Reduction of the number of sequences analyzed allowed for the building of statistically supported phylogenetic trees with bootstrap values >70%. All sequences displaying >98.0% nucleotide identity with the virus sequences generated in this study were included, with the aim to establish nucleotide sequence correlation with highly similar HEV strains from humans, pigs, wild boars, and other animal hosts previously detected in Europe. For sequences provisionally classified within the 3g and 3i subtypes (named here 3g-like and 3l-like), together with unclassified strains, named here 3 ^*∗*^, no sequences fulfilled the established criteria, and phylogenetic analysis was not performed. As shown in Table 2, the number of sequences used to build each dataset, obtained following the criteria described above, is reported. The resulting datasets were used for phylogenetic analysis.

## 3. Results

### 3.1. Identification of HEV Subtypes

**Figure 1:**
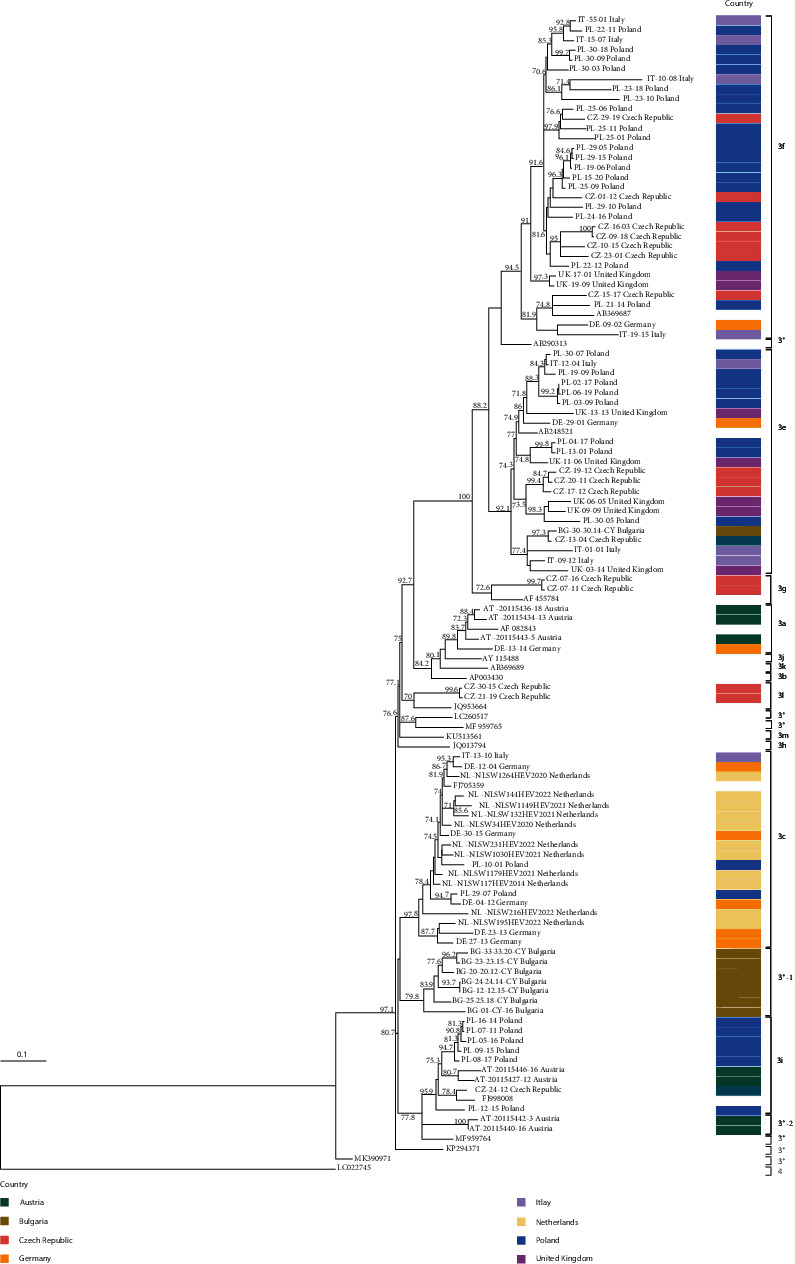
Phylogenetic analysis based on the fragment of the partial ORF2 region of 99 sequences obtained in this study; 18 HEV-3 subtype reference strains were included and one HEV-4 sequence was used as the outgroup. The maximum likelihood tree was built using the TIM model (transition model) with invariant sites and gamma distribution based on 1,000 bootstrap replications, with bootstraps values >70 indicated at their respective nodes. 3 ^*∗*^ indicates unassigned subtypes.

Based on the results of maximum likelihood phylogenetic tree and by HEV-Net typing tool, subtypes were assigned to the 99 nonidentical pig HEV sequences. All sequences belong to HEV-3 and clearly assigned to five subtypes: 3a (4/99, 4.0%), 3c (19/99, 19.2%), 3e (22/99, 22.2%), 3f (32/99, 32.3%), and 3i (9/99, 9.0%). Two sequences clustered with 3g (2/99, 2.0%) and two to 3l (2/99, 2.0%) subtypes albeit showing low nucleotide identities (<89.0% nucleotide identity (nt.id.) with the reference strain sequences (3g, AF455784; 3l, JQ953664) (data not shown). Furthermore, being not classifiable by the HEV-Net typing tool, sequences were considered 3g- and 3l-related (named here 3g-like and 3l-like) and were provisional classified in these subtypes. The remaining nine sequences (9/99, 9.1%), named as 3 ^*∗*^, were not assigned to any currently known subtypes (Figure 1). The nucleotide identities (*p*-distances) for the HEV detected strains, within each cluster and with subtype reference strains for all the subtypes detected in this study, are displayed in *Supplementary 2*.

**Figure 2:**
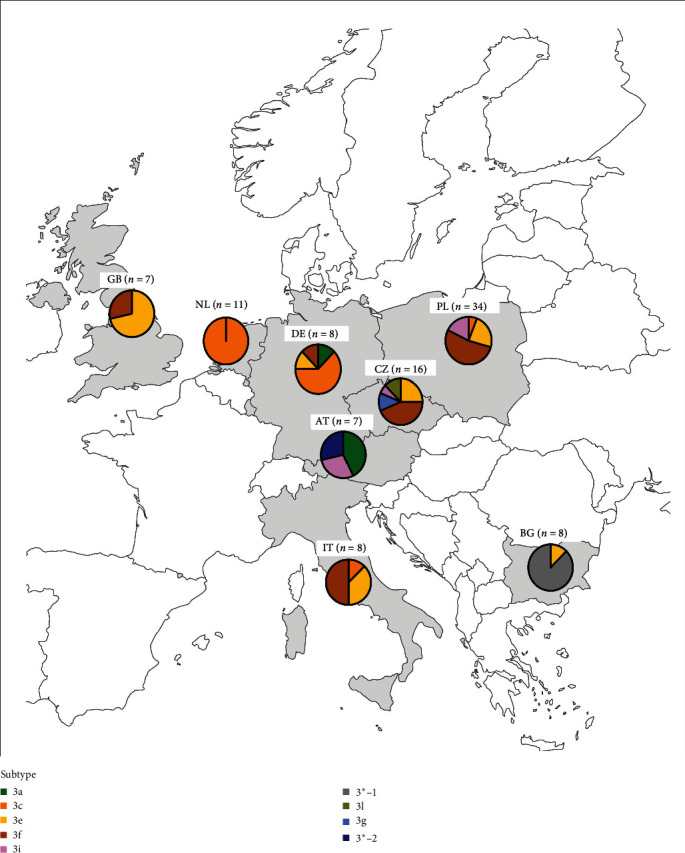
Map of the European countries involved in this study. The participant countries are highlighted in gray. The frequency of the detected HEV subtypes is displayed by pie charts. The number of unique sequences from each country is indicated in round brackets; since one nonidentical sequence was retrieved for each farm, it corresponds to the number of farms. In Czech Republic and in Poland, the number of farms was 15 and 21, respectively, and more nonidentical sequences, total reported in round brackets, were detected within each farm.

The most frequently detected subtype in the considered countries was the 3e (6/8 countries, 75.0%), followed by 3f (5/8, 62.5%) and 3c (4/8, 50.0%). Subtypes circulating less frequently were 3i and 3a, detected in three (3/8, 37.5%) and two countries (2/8, 25.0%), respectively. The subtypes 3g-like and 3l-like were only identified in Czech Republic (1/8, 12.5%) (Table 3). The unassigned sequences formed two different clusters, namely 3 ^*∗*^-1, reported in Bulgaria (1/8, 12.5%), and 3 ^*∗*^-2 in Austria (1/8, 12.5%) (Figures 1 and 2). Since only one strain was found in each farm, the number of sequences from each country was identical to the number of farms, except in Czech Republic and in Poland where more than one nonidentical sequence was obtained from one and six farms, respectively.

The most common subtype among the investigated farms was the 3f, reported in 23 farms, followed by the 3e (21 farms). Subtype 3c was also widespread, being detected in at least eight farms, including those from Netherlands where 3c was detected, but where data on the number of farms sampled are missing. The extent of sequence heterogeneity of strains circulating varied depending on the countries. Sequence analysis showed that identical strains (100% nt.id.) were only detected either within farm (74 farms, 100%) or among farms in the same country (4/74 farms, 5.0%). As shown in Table 3, except for Netherlands where only the 3c subtype was reported, different subtypes were simultaneously circulating in all the investigated countries. Of note is that the 3f subtype, when present, was also predominant at country level being the most common in the analyzed farms in Italy, Czech Republic, and Poland with the frequencies of 50.0%, 46.7%, and 42.8% of the investigated farms, respectively (Table 3). In Germany, the 3c subtype was more circulating, being present in five farms (5/8, 62.5%, Table 3). In three farms in Poland, more than one subtype was observed with the following combinations: 3e and 3f in two farms and 3c and 3f in one farm, while in Czech Republic, two nonidentical 3g-like strains were detected in the same farm (Table 3).

### 3.2. Phylogenetic Analyses and Correlation between Pig and Human Strains

#### 3.2.1. Analyses of Strains Belonging to Subtype 3a

Eleven sequences (11/291, 3.8%) belonged to subtype 3a as revealed by clustering with the reference strain Meng (AF082843) showing 89.0%–92.0% nt.id. (*Supplementary 3*). The four nonidentical sequences were obtained from three farms in Austria and one in Germany. Pig HEV sequences from Austria (AT-20115434−13, AT-20115436−18, AT-20115443−5) were grouped together, and shared 90.4%–96.0% nt.id. in a cluster, including two Austrian strains detected in a previous study on pigs (HM623775, HM623776) and a human strain from Hungary (FJ641051) with nt.id. ranging from 92.0% to 98.0%. The German sequence (DE−13−14) belonged to a different cluster and was grouped with a human strain detected in Hungary (94.0% nt.id., EF530661) and a swine strain from Canada (92.4% nt.id., FJ617438). Overall, pig HEV sequences of 3a subtype from this study showed nucleotide identities ranging between 93.7% and 94.7% with human strains, displaying interhost similarity with strains detected in Hungary.

#### 3.2.2. Analyses of Strains Belonging to Subtype 3c

Forty-two sequences (42/291, 14.4%) resulting in 19 nonidentical sequences from Germany, Italy, Netherlands, and Poland belonged to the 3c subtype, sharing 90.0%–96.0% nt.id. with 3c reference strain wbGER27 (FJ705359) (*Supplementary 4*). No geographical clustering was observed among sequences obtained in this study, being interspersed with other European sequences, even if the 3c subtype was predominant in the North European countries such as Germany and Netherlands and sporadically detected in East (Poland) and South (Italy) side of the involved European countries. The 3c subtype represented the only subtype reported in Netherlands, displaying a high sequence heterogeneity (90.0%–97.0%), with 11 sequences belonging to 11 different clusters. The same heterogeneity was observed for strains detected in German (*n* = 5) and Polish farms (*n* = 2), where each strain belonged to a different cluster. Interstingly, in the Polish farm PL-29 five different HEV strains were detected at the same time, three strains were belonging to the 3f subtype and two to the 3c subtype. The correlation between swine and human 3c sequences was high, with a range of nucleotide identity between 94.9% and 99.8%. This correlation was present in each cluster of the phylogenetic tree, with strong evidence of circulation of highly correlated strains in humans and pigs in the same country, i.e., in Netherlands. Overall, the 3c sequences obtained in this study and those previously detected across Europe showed both interhost and intercountry correlation: the highest nt.id. of 99.8% was observed between the pig strain NL-NLSW132HEV2021 (this study, 2021) and a German strain (MZ814705) detected in humans in 2019.

#### 3.2.3. Analyses of Strains Belonging to Subtype 3e

A total of 59 (59/291, 20.3%) subtype 3e sequences from 21 farms were detected in six countries and, among them, 22 were nonidentical (*Supplementary 5*). Sequences showed nt.id. ranging from 88.0% to 92.0% with the 3e reference strain swJ8-5 (AB248521). It has to be mentioned that none of the sequences from outside EU fulfilled the established criteria for inclusion in the tree. The phylogenetic tree showed that a single sequence (100% nt.id.) was detected on each farm, except for Polish farm PL-30, which presented two different 3e strains, PL−30−05 and PL−30−07, sharing 89.0% nt.id. Strains from the United Kingdom (UK−06−05, UK−09−09), Poland (PL−02−17, PL−06−19, PL−03−09), and Czech Republic (CZ−19−12, CZ−20−11, CZ−17−12) showed a geographic clustering, being more correlated with strains detected within the country. However, a few strains clustered more closely with strains detected from abroad, i.e., BG−30−30.14−CY, Bulgaria, and CZ−13−04, Czech Republic. Some clusters were formed by human and pig HEV strains reported in different countries, i.e., the Italian swine IT−09-12 clustered with a human (MH504149) strain detected in the United Kingdom (97.7% nt.id. each other) and three pig strains from Czech Republic (CZ−17−12, CZ−20−11, CZ−19−12) clustered with a human German (MK089848) strain (97.4%–97.6% nt.id. each other). Some pig strains displayed a lower identity when compared with human strains detected from the same country, i.e., 93.5% nt.id. between the swine DE−29−01 and a human strain (MZ814590) both detected in Germany.

#### 3.2.4. Analyses of Strains Belonging to Subtype 3f

The 3f subtype was the most common (107 total out of 291, 36.8%) with 32 nonidentical sequences from 23 farms located in five countries (*Supplementary 6*). The 3f sequences were grouped in two main clusters, including four and 28 sequences, classified as 3f1 and 3f2, respectively, by HEV-Net database typing tool. Both clusters shared 87.0%–92.0% nt.id. with the 3f reference strain E116-YKH98C (AB369687). The 3f1 cluster included sequences from farms located in Italy (IT-19), Poland (PL-21), Czech Republic (CZ-15), and Germany (DE-09), which showed 90.0%–92.0% nt.id. to each other and were further grouped in different subclusters. The 3f2 cluster included 28 sequences from Czech Republic, Italy, Poland, and the United Kingdom. Among them, 16 from Czech Republic, Poland, and the United Kingdom showed an intracountry clustering. Sequences from Polish farms (PL-15, PL-19, PL-22, PL-23, PL-25, PL-29, PL-30) showed high intrafarm sequence heterogeneity (88.0%–99.0%), with different strains circulating simultaneously in the same farm. Three sequences detected on farm PL-30 were classified as 3f. Two of them (PL-30-09, PL-30-18) shared 98.4% nt.id., while the third sequence (PL-30-03) showed 92.2% and 91.7% nt.id. with the former sequences, respectively. Overall, in the farm PL-30, five different sequences were reported, three belonging to the 3f subtype and two to the 3e subtype. All sequenced strains from this study clustered with other sequences from Europe. For the 3f subtype, the correlation between swine and human strains was highly variable ranging between 92.5% and 98.7% nt.id. The highest correlation of 98.8% nt.id. was observed in Italy between the swine IT−10−08 and the human MN537876.

#### 3.2.5. Analyses of Strains Belonging to Subtype 3i

Thirty-eight 3i sequences were reported from nine farms located in Austria, Czech Republic, and Poland (38/291, 13.0%), and nine resulted in nonidentical sequences sharing 90.0%–92.0% nt.id. with the 3i reference strain BB02 (FJ998008) (*Supplementary 7*). Austrian strains displayed 95.5% nt.id. to each other and clustered with a pig strain previously described in Austria (93.0%–94.0% nt.id., HM623777). The Polish sequences were separated in two clusters sharing 91.7%–93.2% nt.id. Sequence PL-12-15 showed the highest match of 96.0% nt.id. with a wild boar strain from Lithuania (MN545455). Sequence CZ−24−12 was classified as 3i, sharing 92.0% with the 3i reference strain (FJ998008), but clustered out of the group formed by the other 3i strains detected in this study, sharing with them >89.0% nt.id. Based on the criteria of inclusion of sequences in our analyses, only one human sequence (MH377721 Sweden Human) was included in the tree and displayed 92.9%–93.2% nt.id. with a group of Polish sequences, suggesting that this subtype is rare in humans.

#### 3.2.6. Analyses of Strains Belonging to Subtype 3 g-Like

Five sequences (5/291, 1.7%), two of which were nonidentical, were retrieved from a single farm in Czech Republic, and clustered with the 3g reference strain Osh 205 (AF455784) with 87.0% and 88.0% nt.id. Strains CZ-07-11 and CZ-07-16 shared 99.0% nt.id. and the sequence alignment revealed seven different nucleotides over the 493 bp sequenced. However, due to the short region analyzed and to the high *p*-distance displayed with the reference, the subtype assignment is provisional. HEV-Net typing tool could not assign it, and further analyses are required. The closest matching of these sequences was with a human strain reported in Greece (OM654048) with 89.6% nt.id. but classified as 3e and <89.0% with any other sequences by BLASTN searches.

#### 3.2.7. Analyses of Strains Belonging to Subtype 3l-Like

In Czech Republic, eight 3l-like sequences (8/291, 2.7%), of which two were nonidentical (CZ−30−15, CZ−21−19), were identified on two farms. These displayed 99.0% nt.id. to each other and clustered together with the 3l reference sequence FR-SHEV3c-like (JQ953664), sharing 87.4% and 87.2% nt.id., respectively. The HEV-Net typing tool could not assign the sequence to any subtype, even the strains clearly cluster with other 3l but being distant from them. The most similar by BLAST with the NCBI was a human strain (MW355302.1) with 91.2% nt.id. and classified as 3h. This mismatch with our findings could be related to recent assign of the 3l and to the short genome fragment analyzed.

#### 3.2.8. Analyses of Strains Not Assigned to Subtypes (Named 3 ^*∗*^)

For 21 sequences (21/291 7.2%), the subtype was not assigned as they were equally distant from several subtypes. Overall, nine were nonidentical, two were unassigned sequences detected in Austria (named here 3 ^*∗*^-1) and seven in Bulgaria (3 ^*∗*^-2). The cluster including the seven Bulgarian sequences, named 3 ^*∗*^-1, sharing 89.0%–97.0% nt.id. to each other, displayed <92.0% nt.id. with other strains from NCBI. The cluster including the two sequences detected in Austria, named 3 ^*∗*^-2, showed <90.0% nt.id. with the other NCBI HEV sequences, with the same *p*-distances to 3i and to a novel subtype recently identified but not assigned yet (MF959764). The sequences were unassigned by HEV-Net typing tool. Overall, despite the low nucleotide identity with all other sequences, its closest human strain was MW355383.1 with 89.5%–89.9% nt.id.

## 4. Discussion

In this study, we investigated the distribution and genetic variability of HEV in domestic pigs in Europe over more than 74 farms located in eight countries: Austria, Bulgaria, Czech Republic, Germany, Italy, Netherlands, Poland, and the United Kingdom. The HEV genome fragment within the ORF2 selected for sequencing has been previously described as suitable for obtaining a robust subtype attribution and for the phylogenetic analyses [12, 51]. However, when only few sequences belonging to a specific subtype are available in the public databases for comparisons and analyses, the ambiguous results of subtype attribution, as it was for the 3g-like and 3l-like strains detected in this study, need further confirmation. Overall, this study confirmed that HEV-3 is predominant in Europe [5]. The phylogenetic analysis revealed differences and similarities between HEV subtypes circulating in pigs in Europe. Nonidentical but similar strains were detected in different countries, but a geographical distribution of subtypes was observed. The 3c subtype is predominant in Northern Europe, Netherlands, and Germany, while 3i only circulates in the Eastern (Poland, Czech Republic) and Central Europe (Austria). The 3e and 3f subtypes were widely distributed over the seven countries participating in the study. Interestingly, putative novel subtypes were detected in Austria and Bulgaria and were confined to the respective countries of detection. This epidemiological pattern could be due to the recent emergence or introduction of these novel HEV strains in Austria and Bulgaria, highlighting the need of a constant future monitoring to evaluate the spread to other countries. It is noteworthy that the top tree subtypes (3c, 3e, and 3f) detected in this study are in agreement with those obtained in previous studies, exploring genetic variability of pig and human HEV strains in Europe [33–37, 39, 52–55]. The dominance of subtypes 3c, 3e, and 3f in humans and pigs could be caused by either a better adaptation to the main reservoirs, humans and pigs, or by an older introduction of these subtypes in Europe [56] and subsequent wider spreading over the continent. Similarly, besides the global spreading of HEV-3 [29], some geographical demarcation at continental level can be observed. The phylogenetic analyses revealed that subtypes 3e, 3f, and 3g are mainly represented by animal and human sequences from Europe, while subtypes 3a and 3c include sequences detected worldwide, and the 3i consists of a limited number of only European sequences from animal host (wild and domestic pigs). This study highlighted the spread and circulation of HEV subtypes in pigs across Europe. The import/export of pigs has been proven to determine the spread of the virus subtypes from one country to another and within the same country [57]. Our findings may corroborate this dynamic of spreading, since highly related HEV strains were detected from pigs housed in different European countries. For instance, pig sequences belonging to the 3c subtype, predominant in North countries, were correlated with other human and pig sequences detected in several European countries. In this study, identical strains were found on different farms in the same country (Bulgaria, Poland, and Czech Republic). This could be linked to the pyramidal structure of commercial pig farming and the movement of animals between farms such as a nucleus breeding site-sending gilts to many commercial breeding farms and, then, each breeding farm distributing weaners to several fattening units, which lead to the spread of the same virus down the pig production pyramid. Transportation of pigs by trucks between farms can also contribute to the spread of the virus if vehicles (particularly wheels) are not properly cleaned [57].

A unique HEV strain was detected in 67 out of 74 farms. The presence and persistence of the same unique strain on pig farms over a long time have been previously described [53, 58] and linked to the ability of the virus to persist in the environment. Conversely, on six farms in Poland, more strains and subtypes were sequenced and coexist on each farm, with up to four nonidentical strains identified, belonging to two different subtypes: 3e and 3f or 3c and 3f. Animals mixing at fattening unit, which originated from different barns, could explain this. However, the presence of different strains may also be linked to the introduction of newly infected animals from other farms. It is remarkable that the largest number of sequences (121) was obtained from Poland that could have contributed to the observed results. Similarly, one farm in Czech Republic displayed the presence of two distinct 3g-like HEV strains. The 3g subtype was originally retrieved from a pig in Kyrgyzstan and it has only been detected in Czech Republic in humans [59], wild boars, and pigs [7, 59–61]. It is interesting that this 3g-like strain has never been detected outside Czech Republic, even many years after it was first detected. This could be due to both the lack of subtyping data and the characteristics of the national trade of pigs, as Czech Republic has very limited export of live pigs. Another interesting subtype distribution was observed in Netherlands, where only the 3c subtype was found to be circulating in pigs in this study and is prevalent in humans [35]. After Denmark, Netherlands is the main exporter of live pigs in the EU and mainly to border countries (https://www.pig333.com/articles/present-and-future-of-live-pig-trade-in-the-eu_18505/), such as Germany where 3c was also widely detected. This could account for the circulation of only one subtype in the country, with no importation of novel strains from abroad since live pigs are not introduced. For Austria and Bulgaria, this is the first subgenotyping report. Results highlighted a high variability in the detected strains belonging to several subtypes. Previous studies reported the circulation of HEV-3 in both countries in humans [10, 62], but information on subtypes is lacking from Austria and limited from Bulgaria, where 3e was previously retrieved in both humans and pigs [63]. Two possible novel HEV-3 subtypes were detected in these countries and they did not spread abroad. Further studies, including full genome sequencing of unassigned strains needed for subtype attribution, will shed more light on HEV occurrence and subtype circulation in Austrian and Bulgarian pigs. A similar degree of variability was observed in Poland, where results of subtyping in pigs were reported for the first time and coincided with 3f and 3e subtypes detected in the production chain of offal-derived pork foodstuffs [64] and in the sole study on human Polish blood donors [65]. In contrary to this finding, Polish population of wild boars seems to have slightly different pool of infecting subtypes with respect to those detected in pigs [66], except for the 3i strains, which were also found in pigs in this study. It is highly likely that a broader movement and mutual transmission of HEV-3 strains between wild boars and pigs were not frequent due to introduced ASF-related biosecurity measures on Polish pig farms. Our results on the prevalence of the HEV-3 subtypes reflect the situation of HEV infections in humans in Europe and the predominance of 3c in Germany [34] and in Netherlands [35] in both pigs and humans. Although HEV is known to be very heterogeneous, it was surprising that there was none of the HEV-3c strain from pigs in this study showing 100% nt.id. with a human HEV-3c strain. The lack of mutual sequence identity could be explained by the higher genetic divergence observed among HEV-3c strains compared to other virus subtypes. Likewise, the low number of available HEV sequences of human origin for phylogenetic analyses could also be responsible for the lack of observed sequence similarity.

In the United Kingdom, the 3c subtype was not detected in pigs, even if it represents the major circulating subtype in humans in that country. In the United Kingdom, most pork is imported from mainland Europe, and this import could account for the detection of different subtypes, as observed in pigs and humans in UK in this and in previous studies [31, 32, 67]. In Italy, results obtained in this study confirmed previous findings, with the 3e and 3f subtypes having a higher circulation than the 3c in both pigs and in humans [37]. Detection of identical sequences in humans and pigs is rare but analyzing the single cluster of strains belonging to the same subtypes, there are many high correlated strains in terms of nucleotide identities (>97% nt.id.) and belonging to both hosts. Highly correlated stains are more frequent in the 3c subtype, may be for a bias because many human HEV sequences are greatly reported. This observation also applies to the 3e and 3f clusters. In Italy, the presence of strains from pigs, wild boar, and humans sharing 99.9%–100% nt.id. confirmed the role of sequencing in tracing the link between human and animal strains and in enhancing the preparedness for possible spillover events of pathogens between animal species and humans.

Subsequent sequence analysis of HEV-3 strains detected in this study confirmed the circulation of the same subtypes in pigs and wild boar. However, the genetic variability observed in pigs is more limited than in wild boar where a higher number of subtypes have been described [25, 29]. The role of wild boar as reservoir of different HEV-3 subtypes deserves a further investigation due to the observed strain variability hosted by this animal species [29]. On the other hand, the presence of zoonotic HEV strains carried by wild boar proves their importance in the epidemiology of HEV infections both in animals and in humans [68, 69].

Three possible limitations were identified in this study: (1) an underestimation of HEV subtype prevalence in each country due to testing of pooled fecal samples; (2) sequencing of different HEV strains potentially present in the same pooled sample and their subsequent assignment to a unique consensus sequence. However, this limitation is mitigated by the use of PCR and Sanger sequencing, which enable the amplification and sequencing of the predominant virus strains present in a sample; and (3) the disproportion in the numbers of analyzed farms in each country, which may not be representative for the studied animal population. Despite this, the results obtained still clearly demonstrated the genetic diversity of the detected virus strains, as well as country-related specific distribution pattern of the HEV subtypes.

## 5. Conclusions

This study showed the genetic diversity of HEV-3 strains circulating on pig farms across Europe. The molecular analysis performed represents results on HEV-3 subtype identification obtained in a relatively short time span (3 years) and confirms the wide heterogeneity of HEV-3 strains present in domestic pigs. The high correlation between humans and pig strains provided further evidence for their possible zoonotic transmission to humans. The study confirmed the circulation of several subtypes in pig host, as well as the existence of novel subtypes, which have not been characterized yet. The monitoring of subtypes circulation and their characterization is important, since different subtypes may play a role in the severity of the disease in humans [34, 40, 70]. The results obtained may suggest that HEV-3 strains have spread from one country to another or within a country as a consequence of both, the trade-related movement of infected domestic pigs and the transboundary movement of wild boars. Based on this result, HEV surveillance is needed to trace strains circulation among countries to evaluate the spreading of novel strains and to identify the source of infection in human cases. Additionally, knowledge on HEV subtypes detected in pigs may not have only epidemiological but also food safety significance for public health.

## Figures and Tables

**Table 1:** Summary of samples sequenced obtained from each country and number of farms investigated.

Country	Number of sequences	Number of farms
Austria	23	7
Bulgaria	12	8
Czech Republic	56	15
Germany	19	8
Italy	23	8
Netherlands	22	NA
Poland	121	21
The United Kingdom	15	7
Total	291	74

*Note*: NA, not available.

**Table 2:** Number of sequences included in the datasets and used for the phylogenetic analysis.

Subtype	This study	NCBI sequences ^*∗*^	HEV references ^*∗∗*^	Total
3a	4	49	19	72
3c	19	133	19	171
3e	22	46	19	87
3f	32	103	19	154
3i	9	7	19	35

*Note*:  ^*∗*^Number of sequences that matched by BLASTN and retrieved from NCBI database.  ^*∗∗*^18 HEV-3 subtype reference sequences and one HEV-4 sequence as outgroup.

**Table 3:** Summary of HEV subtypes identified for each country.

Subtype	Country								Total
	Austria	Bulgaria	Czech Republic	Germany	Italy	Netherlands	Poland	United Kingdom	
3a	3/9 (3)			1/2 (1)					4/11 (4)
3c				5/8 (5)	1/3 (1)	11/22^a^	1/6 (1)		18/39 (7)
3c + 3f							1/3 + 3/9 (1)^b^		4/12 (1)^b^
3e		1/1 (1)	4/8 (4)	1/2 (1)	3/11 (3)		4/20 (5)	5/9 (5)	18/51 (19)
3e + 3f							4/8 + 3/6 (2)^b^		7/14 (2)^b^
3f			7/30 (7)	1/7 (1)	4/9 (4)		12/40 (6)	2/6 (2)	26/92 (21)
3g-like			2/5 (1)						2/5 (1)
3i	2/4 (2)		1/5 (1)				6/29 (6)		9/38 (9)
3l-like			2/8 (2)						2/8 (2)
3 ^*∗*^^c^	2/10 (2)	7/11 (7)							9/21 (9)
**Total**	**7/23 (7)**	**8/12 (8)**	**16/56 (15)**	**8/19 (8)**	**8/23 (8)**	**11/22^a^**	**34/121 (21)**	**7/15 (7)**	**99/291 (74)**

*Note*: Data are reported as number of sequences nonduplicated/total; number of farms are indicated in round brackets. ^a^Data on farms are not available. ^b^Farms with several subtypes detected. ^c^Sequenced strains belonging to different subtypes but not assignable to any of the subtypes defined so far (namely 3 ^*∗*^).

## Data Availability

The data that support the findings of this study are available from the authors upon reasonable request.

## Supplementary Materials

Supplementary 1 Sequences of HEV-3 strains detected in pigs across Europe and included in the phylogenetic trees as unique sequences.

Supplementary 2 Nucleotide identities (*p*-distance) displayed by detected strains within each cluster and with subtype reference strains.

Supplementary 3 Maximum likelihood phylogenetic tree built on short genome fragment of the partial ORF2 region of 72 HEV-3a sequences: four from this study, 49 from NCBI database, 18 HEV-3 subtype reference strains, and one HEV-4 sequence used as outgroup.

Supplementary 4 Maximum likelihood phylogenetic tree built on short genome fragment of the partial ORF2 region of 171 HEV-3c sequences: 19 from this study, 133 from NCBI database, 18 HEV-3 subtype reference strains, and one HEV-4 sequence used as outgroup.

Supplementary 5 Maximum likelihood phylogenetic tree built on short genome fragment of the partial ORF2 region of 87 HEV-3e sequences: 22 from this study, 46 from NCBI database, 18 HEV-3 subtype reference strains, and one HEV-4 sequence used as outgroup.

Supplementary 6 Maximum likelihood phylogenetic tree built on short genome fragment of the partial ORF2 region of 154 HEV-3f sequences: 32 from this study, 103 from NCBI database, 18 HEV-3 subtype reference strains, and one HEV-4 sequence used as outgroup.

Supplementary 7 Maximum likelihood phylogenetic tree built on short genome fragment of the partial ORF2 region of 35 HEV-3i sequences: nine from this study, seven from NCBI database, 18 HEV-3 subtype reference strains, and one HEV-4 sequence used as outgroup.
