# Supplementary material for: Molecular Characterization and Phylogenetic Analysis of Hepatitis E Virus (HEV) Strains from Pigs Farmed in Eight European Countries between 2020 and 2022
Source: Transbound Emerg Dis. 2023 Dec 7;2023:2806835. doi: 10.1155/2023/2806835 (PMC12016832; doi:10.1155/2023/2806835)
Supplement: Supplementary 1 — Sequences of HEV-3 strains detected in pigs across Europe and included in the phylogenetic trees as unique sequences. [file 2806835.f1.docx]

**Supplementary table 1.** Sequences of HEV-3 strains detected in pigs across Europe and included in the phylogenetic trees as unique sequences.

| **Sequence ID** | **Country** | **Farm ID** | **Date of sampling** | **HEV-3 subtype** |
| --- | --- | --- | --- | --- |
| **AT-20115427-12** |  | AT_427 | 2020-10-05 | 3i |
| **AT-20115434-13** |  | AT_434 | 2020-10-05 | 3a |
| **AT-20115436-18** |  | AT_436 | 2020-10-05 | 3a |
| **AT-20115440-16** | Austria | AT_440 | 2020-10-05 | 3*-2 |
| **AT-20115442-3** |  | AT_442 | 2020-10-05 | 3*-2 |
| **AT-20115443-5** |  | AT_443 | 2020-10-05 | 3a |
| **AT-20115446-16** |  | AT_446 | 2020-10-05 | 3i |
| **BG-01-CY-19** |  | BG_1 | 2020-10-20 | 3*-1 |
| **BG-12-12.15-CY** |  | BG_12 | 2021-04-21 | 3*-1 |
| **BG-20-20.12-CY** |  | BG_20 | 2021-05-12 | 3*-1 |
| **BG-23-23.15-CY** | Bulgaria | BG_23 | 2021-05-19 | 3*-1 |
| **BG-24-24.14-CY** |  | BG_24 | 2021-05-19 | 3*-1 |
| **BG-25-25.18-CY** |  | BG_25 | 2021-06-08 | 3*-1 |
| **BG-30-30.14-CY** |  | BG_30 | 2021-06-22 | 3e |
| **BG-33-33.20-CY** |  | BG_33 | 2021-06-22 | 3*-1 |
| **CZ-01-12** |  | CZ_1 | 2020-10-05 | 3f |
| **CZ-07-11** |  | CZ_7^#^ | 2020-11-06 | 3g-like |
| **CZ-07-16** |  | CZ_7^#^ | 2020-11-06 | 3g-like |
| **CZ-09-18** |  | CZ_9 | 2020-11-16 | 3f |
| **CZ-10-15** |  | CZ_10 | 2020-11-17 | 3f |
| **CZ-13-04** |  | CZ_13 | 2020-11-20 | 3e |
| **CZ-15-17** |  | CZ_15 | 2020-11-27 | 3f |
| **CZ-16-03** | Czech Republic | CZ_16 | 2020-12-02 | 3f |
| **CZ-17-12** |  | CZ_17 | 2021-01-18 | 3e |
| **CZ-19-12** |  | CZ_19 | 2021-01-22 | 3e |
| **CZ-20-11** |  | CZ_20 | 2021-02-02 | 3e |
| **CZ-21-19** |  | CZ_21 | 2021-02-17 | 3l-like |
| **CZ-23-01** |  | CZ_23 | 2021-03-16 | 3f |
| **CZ-24-12** |  | CZ_24 | 2021-03-18 | 3i |
| **CZ-29-19** |  | CZ_29 | 2021-04-21 | 3f |
| **CZ-30-15** |  | CZ_30 | 2021-04-23 | 3l-like |
| **DE-04-12** |  | DE_4 | 2021-03-30 | 3c |
| **DE-09-02** |  | DE_9 | 2021-02-23 | 3f |
| **DE-12-04** |  | DE_12 | 2021-03-23 | 3c |
| **DE-13-14** | Germany | DE_13 | 2021-03-23 | 3a |
| **DE-23-13** |  | DE_23 | 2020-11-26 | 3c |
| **DE-27-13** |  | DE_27 | 2021-01-18 | 3c |
| **DE-29-01** |  | DE_29 | 2021-04-22 | 3e |
| **DE-30-15** |  | DE_30 | 2021-04-13 | 3c |
| **IT-01-1** |  | IT_1 | 2021-05-05 | 3e |
| **IT-09-12** |  | IT_9 | 2020-02-08 | 3e |
| **IT-10-8** |  | IT_10 | 2020-11-19 | 3f |
| **IT-12-13** | Italy | IT_12 | 2021-01-08 | 3e |
| **IT-13-10** |  | IT_13 | 2021-03-04 | 3c |
| **IT-15-7** |  | IT_15 | 2021-02-17 | 3f |
| **IT-19-15** |  | IT_19 | 2021-07-07 | 3f |
| **IT-55-1-1** |  | IT-55 | 2021-10-10 | 3f |
| **NL-NLSW1030HEV2021** |  | NL-1 | 2020-10-04 | 3c |
| **NL-NLSW1149HEV2021** |  | NL-2 | 2021-03-17 | 3c |
| **NL-NLSW1179HEV2021** |  | NL-3 | 2021-04-20 | 3c |
| **NL-NLSW117HEV2022** |  | NL-4 | 2022-01-20 | 3c |
| **NL-NLSW1264HEV2020** |  | NL-5 | 2020-11-06 | 3c |
| **NL-NLSW132HEV2021** | The Netherlands | NL-6 | 2021-03-11 | 3c |
| **NL-NLSW144HEV2022** |  | NL-7 | 2021-04-15 | 3c |
| **NL-NLSW195HEV2022** |  | NL-8 | 2022-01-08 | 3c |
| **NL-NLSW216HEV2022** |  | NL-9 | 2022-01-16 | 3c |
| **NL-NLSW231HEV2022** |  | NL-10 | 2022-01-17 | 3c |
| **NL-NLSW34HEV2020** |  | NL-11 | 2020-10-04 | 3c |
| **PL-02-17** |  | PL_2 | 2021-02-03 | 3e |
| **PL-03-9** |  | PL_3 | 2021-02-03 | 3e |
| **PL-04-17** |  | PL_4 | 2021-02-11 | 3e |
| **PL-05-16** |  | PL_5 | 2021-03-15 | 3i |
| **PL-07-11** |  | PL_7 | 2021-03-24 | 3i |
| **PL-08-17** |  | PL_8 | 2021-03-22 | 3i |
| **PL-09-15** |  | PL_9 | 2021-03-22 | 3i |
| **PL-10-01** |  | PL_10 | 2021-03-08 | 3c |
| **PL-12-15** |  | PL_12 | 2021-03-16 | 3i |
| **PL-13-01** |  | PL_13 | 2021-03-17 | 3e |
| **PL-15-20** |  | PL_15 | 2021-04-20 | 3f |
| **PL-16-14** |  | PL_16 | 2021-04-14 | 3i |
| **PL-19-06** | Poland | PL_19^#^ | 2021-04-13 | 3f |
| **PL-19-09** |  | PL_19^#^ | 2021-04-13 | 3e |
| **PL-21-14** |  | PL_21 | 2021-04-26 | 3f |
| **PL-22-11** |  | PL_22^#^ | 2021-04-28 | 3f |
| **PL-22-12** |  | PL_22^#^ | 2021-04-28 | 3f |
| **PL-23-10** |  | PL_23 | 2021-05-05 | 3f |
| **PL-23-18** |  | PL_23 | 2021-05-05 | 3f |
| **PL-24-16** |  | PL_24 | 2021-06-09 | 3f |
| **PL-25-01** |  | PL_25^#^ | 2021-06-09 | 3f |
| **PL-25-06** |  | PL_25^#^ | 2021-06-09 | 3f |
| **PL-25-09** |  | PL_25^#^ | 2021-06-09 | 3f |
| **PL-25-11** |  | PL_25^#^ | 2021-06-09 | 3f |
| **PL-29-05** |  | PL_29^#^ | 2021-06-16 | 3f |
| **PL-29-07** |  | PL_29^#^ | 2021-06-16 | 3c |
| **PL-29-10** |  | PL_29^#^ | 2021-06-16 | 3f |
| **PL-29-15** |  | PL_29^#^ | 2021-06-16 | 3f |
| **PL-30-03** |  | PL_30^#^ | 2021-06-21 | 3f |
| **PL-30-05** | Poland | PL_30^#^ | 2021-06-21 | 3e |
| **PL-30-07** |  | PL_30^#^ | 2021-06-21 | 3e |
| **PL-30-09** |  | PL_30^#^ | 2021-06-21 | 3f |
| **PL-30-18** |  | PL_30^#^ | 2021-06-21 | 3f |
| **PL-6-19** |  | PL_6 | 2021-03-15 | 3e |
| **UK-03-14** |  | UK_3 | 2020-10-13 | 3e |
| **UK-06-05** |  | UK_6 | 2021-06-16 | 3e |
| **UK-09-09** |  | UK_9 | 2021-07-23 | 3e |
| **UK-11-06** | United Kingdom | UK_11 | 2021-10-18 | 3e |
| **UK-13-13** |  | UK_13 | 2021-10-01 | 3e |
| **UK-17-01** |  | UK_17 | 2021-10-14 | 3f |
| **UK-19-09** |  | UK_19 | 2021-10-14 | 3f |

^#^ CZ and PL farms displaying the presence of multiple HEV-3 strains
