# Supplementary material for: Molecular Characterization and Phylogenetic Analysis of Hepatitis E Virus (HEV) Strains from Pigs Farmed in Eight European Countries between 2020 and 2022
Source: Transbound Emerg Dis. 2023 Dec 7;2023:2806835. doi: 10.1155/2023/2806835 (PMC12016832; doi:10.1155/2023/2806835)
Supplement: Supplementary 2 — Nucleotide identities (p-distance) displayed by detected strains within each cluster and with subtype reference strains. [file 2806835.f2.docx]

**Supplementary table 2.** Nucleotide identities (p-distances) displayed by detected strains, within each cluster and with subtype reference strains.

| **Country** |  | **Subtype (%)** | | | | | | | | |
| --- | --- | --- | --- | --- | --- | --- | --- | --- | --- | --- |
|  |  | **3a** | **3c** | **3e** | **3f** | **3g-like** | **3i** | **3l-like** | **3*-1** | **3*-2** |
| **Austria** | Subtype^a^ | 92-98 |  |  |  |  | 91-94 |  |  | 99 |
|  | Reference^b^ | 91-92 |  |  |  |  | 90-92 |  |  | 87-89^c^ |
| **Bulgaria** | Subtype |  |  | - |  |  |  |  | 89-90 |  |
|  | Reference |  |  | 90 |  |  |  |  | 88-90^d^ |  |
| **Czech Republic** | Subtype |  |  | 88 | 87-99^e^ | 99 | - | 99 |  |  |
|  | Reference |  |  | 91 | 87-88 | 87-88 | 92 | 87 |  |  |
| **Germany** | Subtype | - | 90-95 | - | - |  |  |  |  |  |
|  | Reference | 89 | 90-96 | 91 | 93 |  |  |  |  |  |
| **Italy** | Subtype |  | - | 88-91 | 87-95 |  |  |  |  |  |
|  | Reference |  | 95 | 88-92 | 88-89 |  |  |  |  |  |
| **The Netherlands** | Subtype |  | 89-97 |  |  |  |  |  |  |  |
|  | Reference |  | 91-96 |  |  |  |  |  |  |  |
| **Poland** | Subtype |  | 91 | 88-96 | 86-99 |  | 92-99 |  |  |  |
|  | Reference |  | 95-96 | 88-91 | 87-92 |  | 90-91 |  |  |  |
| **United Kingdom** | Subtype |  |  | 86-94 | 97 |  |  |  |  |  |
|  | Ref |  |  | 88-91 | 89-90 |  |  |  |  |  |

- One sequence only reported.

^a^ Nucleotide distances calculated between sequences from this study.

^b^ Nucleotide distances calculated with the reference sequences.

^c^ Closer reference sequence: MF959764, unassigned by Smith et al., 2020.

^d^ Closer reference sequence: FJ705359, 3c assigned by Smith et al., 2020.

^e^Provisionally assigned to two sub-clusters name 3f1 and 3f2 by HEV-Net typing tool.
