# Supplementary figures and images for: Molecular Characterization and Phylogenetic Analysis of Hepatitis E Virus (HEV) Strains from Pigs Farmed in Eight European Countries between 2020 and 2022
Source: Transbound Emerg Dis. 2023 Dec 7;2023:2806835. doi: 10.1155/2023/2806835 (PMC12016832; doi:10.1155/2023/2806835)

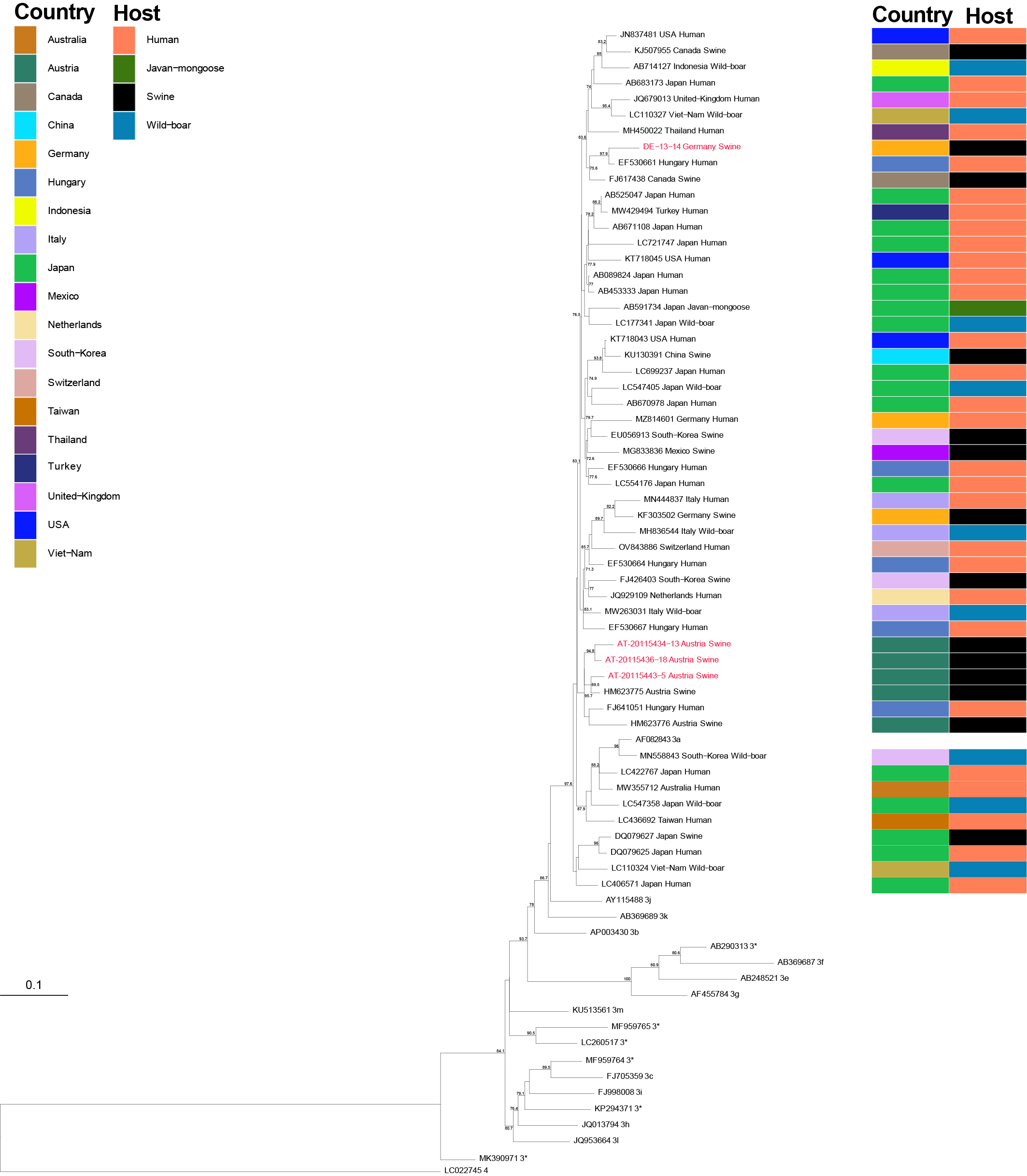

Supplement: Supplementary 3 — Maximum likelihood phylogenetic tree built on short genome fragment of the partial ORF2 region of 72 HEV-3a sequences: four from this study, 49 from NCBI database, 18 HEV-3 subtype reference strains, and one HEV-4 sequence used as outgroup. [file 2806835.f3.jpg]

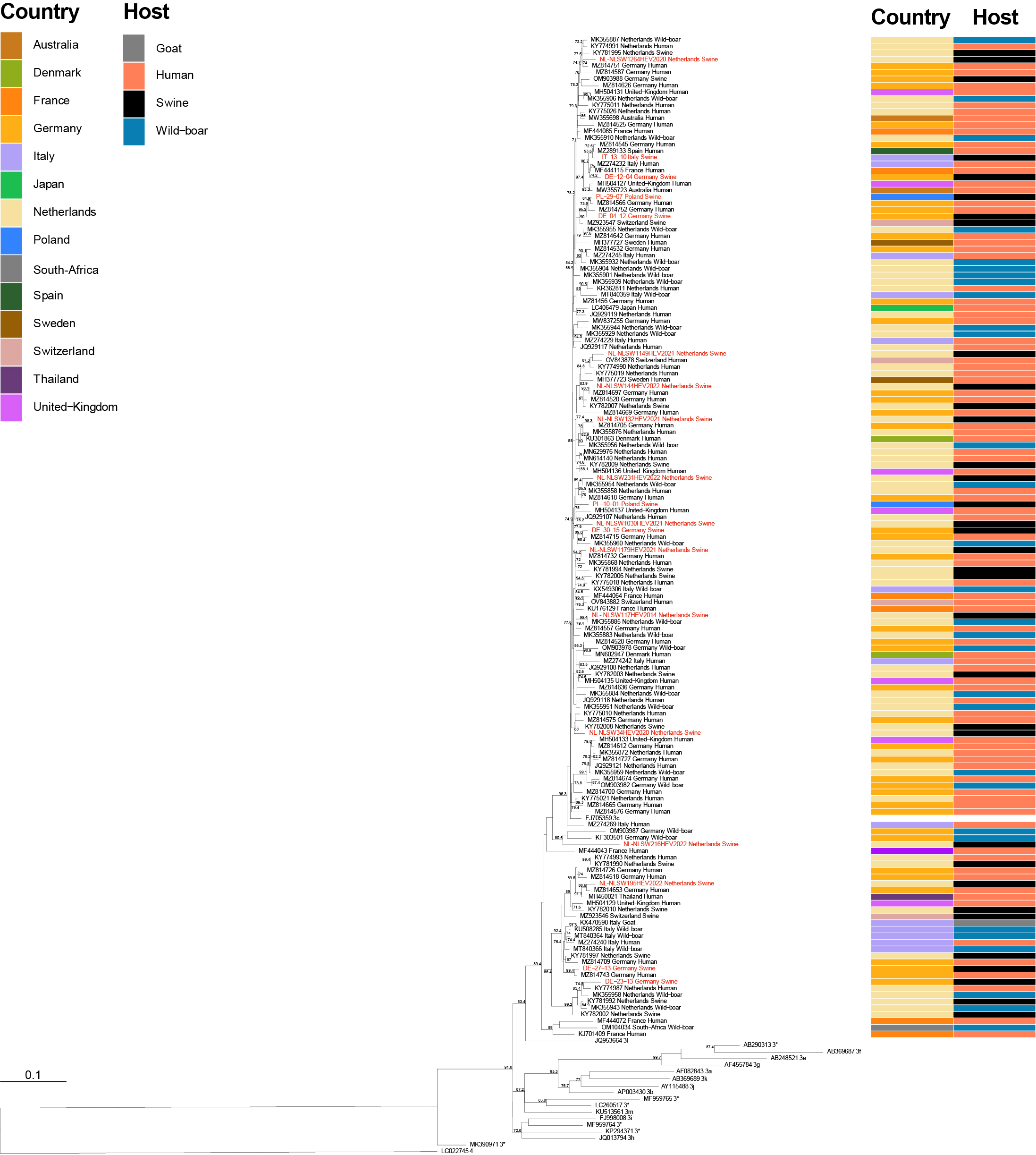

Supplement: Supplementary 4 — Maximum likelihood phylogenetic tree built on short genome fragment of the partial ORF2 region of 171 HEV-3c sequences: 19 from this study, 133 from NCBI database, 18 HEV-3 subtype reference strains, and one HEV-4 sequence used as outgroup. [file 2806835.f4.jpg]

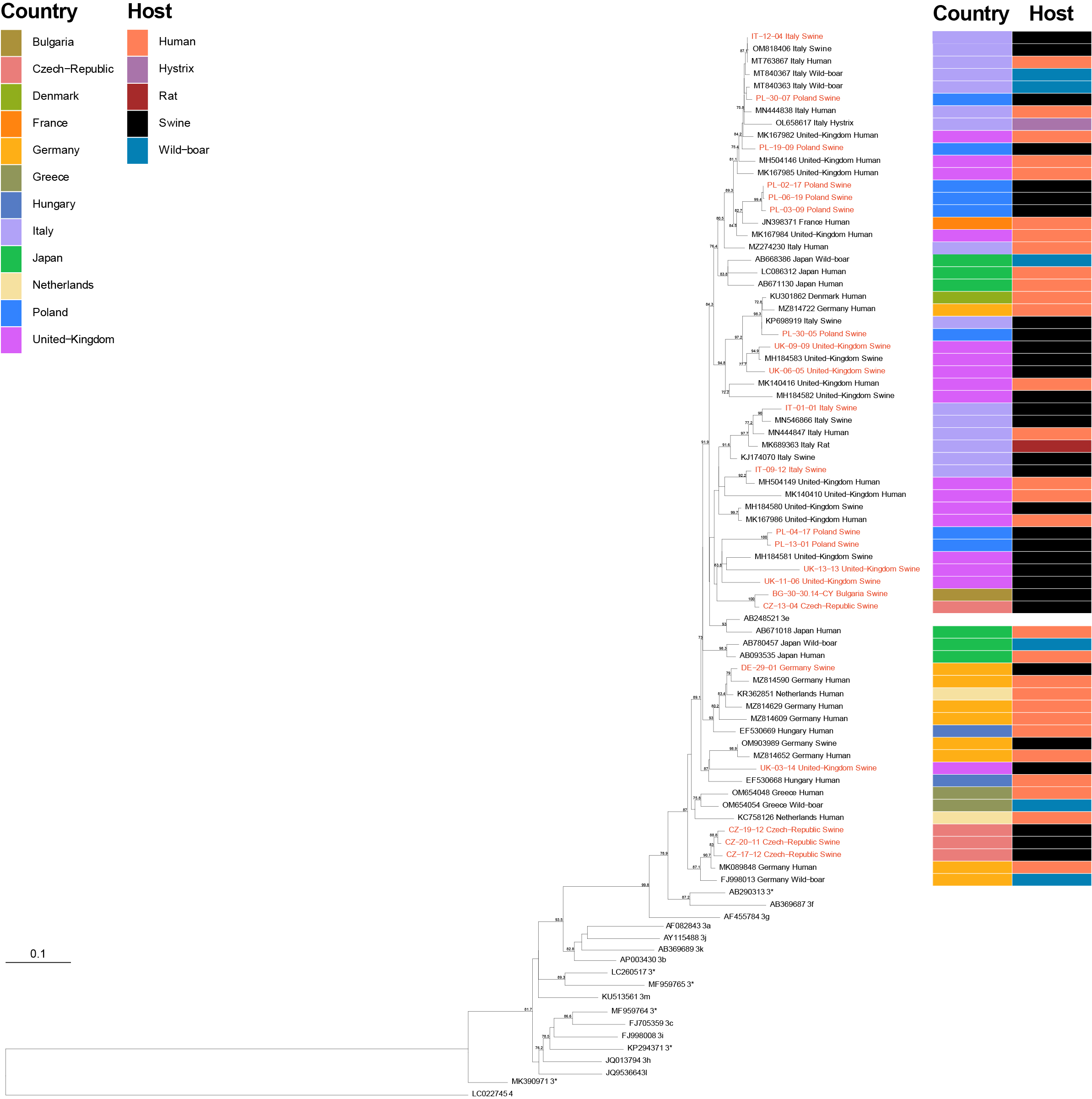

Supplement: Supplementary 5 — Maximum likelihood phylogenetic tree built on short genome fragment of the partial ORF2 region of 87 HEV-3e sequences: 22 from this study, 46 from NCBI database, 18 HEV-3 subtype reference strains, and one HEV-4 sequence used as outgroup. [file 2806835.f5.jpg]

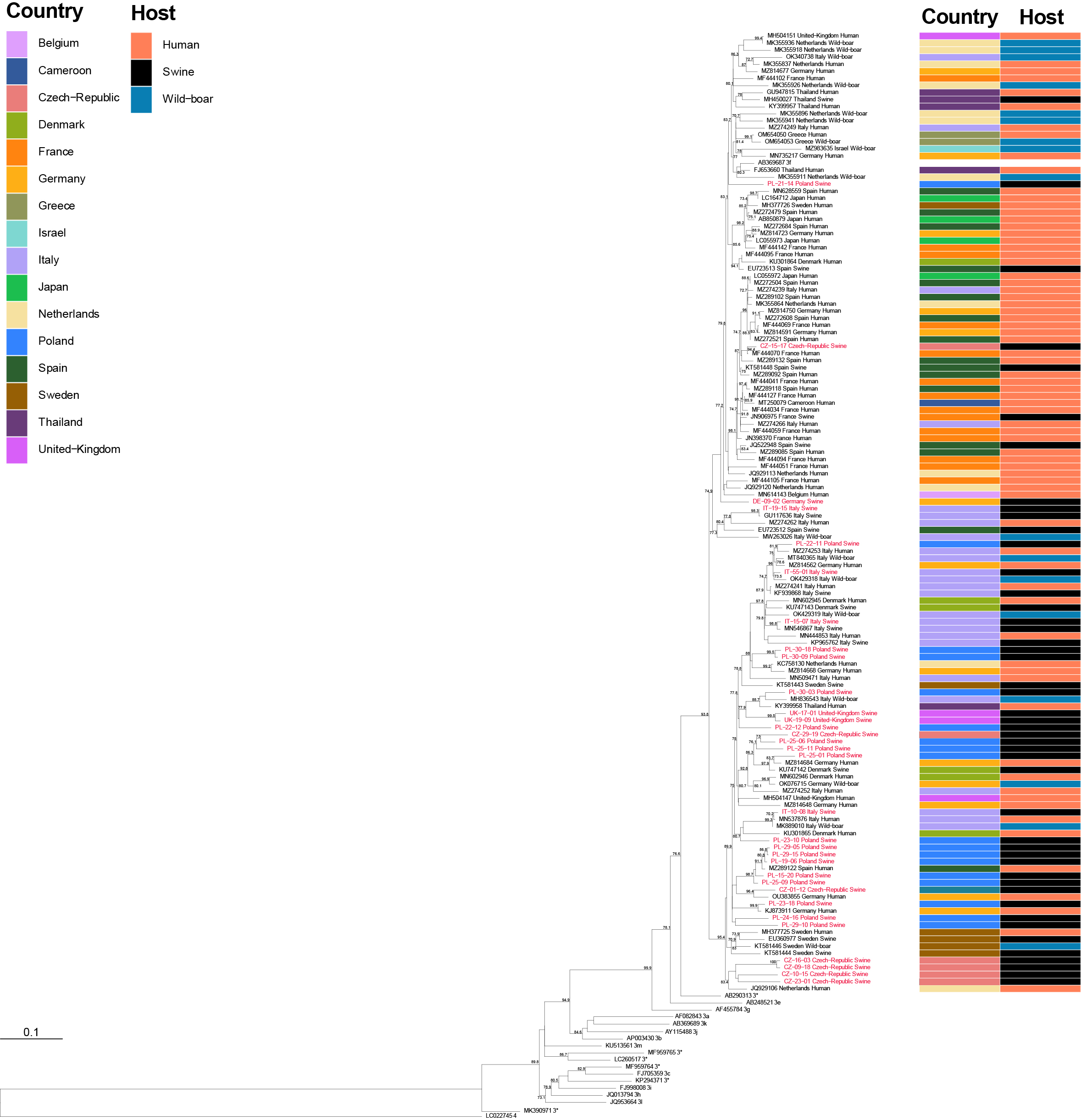

Supplement: Supplementary 6 — Maximum likelihood phylogenetic tree built on short genome fragment of the partial ORF2 region of 154 HEV-3f sequences: 32 from this study, 103 from NCBI database, 18 HEV-3 subtype reference strains, and one HEV-4 sequence used as outgroup. [file 2806835.f6.jpg]

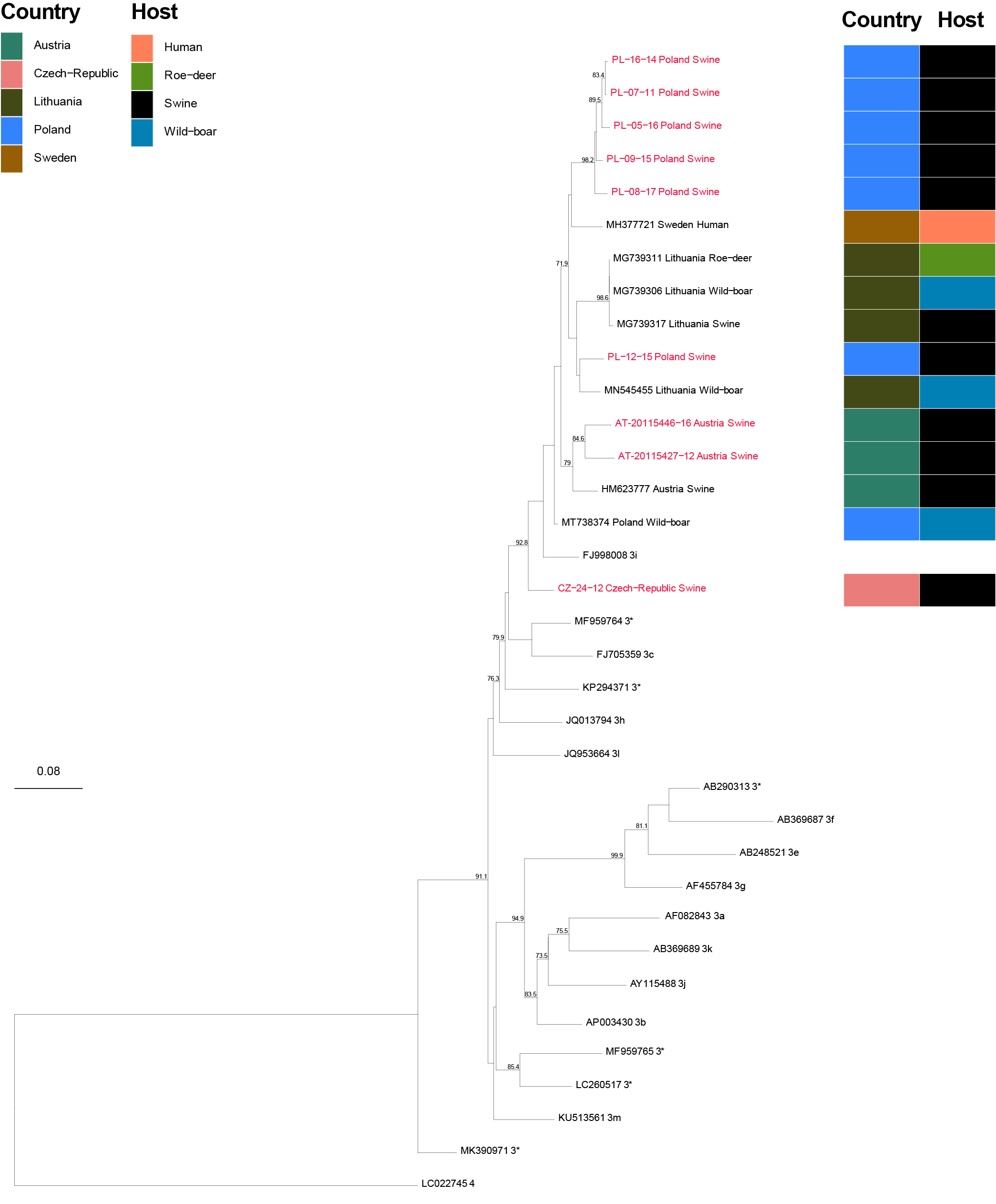

Supplement: Supplementary 7 — Maximum likelihood phylogenetic tree built on short genome fragment of the partial ORF2 region of 35 HEV-3i sequences: nine from this study, seven from NCBI database, 18 HEV-3 subtype reference strains, and one HEV-4 sequence used as outgroup. [file 2806835.f7.jpg]
